# Supplementary material for: Expanding research impact through engaging the maker community and collaborating with digital content creators
Source: PLoS One. 2024 May 8;19(5):e0302449. doi: 10.1371/journal.pone.0302449 (PMC11078436; doi:10.1371/journal.pone.0302449)
Supplement: S1 File — Figure A. Outcomes-focused project logic model which includes 1) Rationale/Needs: Our desired vision and what we hope our actions will result in, 2) Inputs: People, time, funds, and other resources dedicated to the success of the project, 3) Activities: The actions that are taken to achieve desired results, 4) Outputs: tangible direct products of project activities, and 5) Outcomes: Impacts and changes expected as a result of the project. Table A. Total downloads and views statistics for the fifty-seven 3D-printable models provided by maker repository analytics, retrieved the week of October 22, 2023. Table B. Total views statistics for the eight “Instructables” provided by Instructables analytics. (ZIP) [file pone.0302449.s001.zip › S1 File.pdf]

1 Supplemental Material

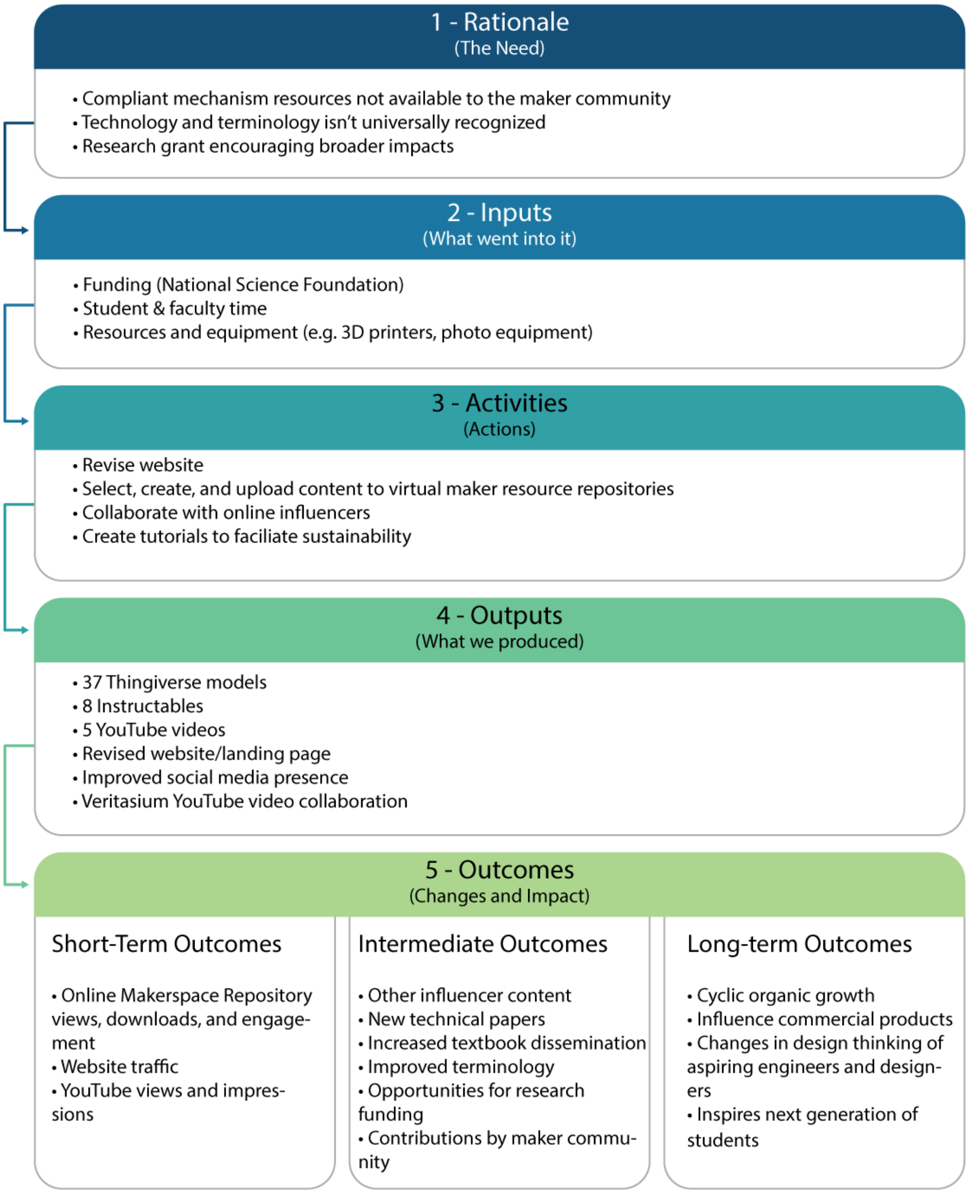

Supplemental Figure 1: Outcomes-focused project logic model which includes *1) Rationale/Needs:* Our desired vision and what we hope our actions will result in, *2) Inputs:* People, time, funds, and other resources dedicated to the success of the project, *3) Activities:* The actions that are taken to achieve desired results, *4) Outputs:* tangible direct products of project activities, and *5) Outcomes:* Impacts and changes expected as a result of the project.

Supplemental Table 1: Total downloads and views statistics for the fifty-seven 3D-printable models provided by maker repository analytics, retrieved the week of October 22, 2023.

“Thing”		Date Published	Views	Downloads	“Thing”		Date Published	Views	Downloads
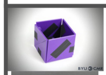	Collapsing Cube	2023 - Oct	106	7	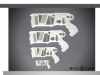	One Piece Compliant Blaster	2023 - Sep	300,750	88,669
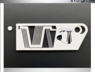	One Piece Compliant Mechanism Disk Launcher	2023 - Sep	45,780	7,228	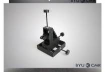	Micromanipulator	2023 - Sep	10,021	344
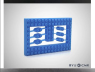	LEGO Compatible Bistable Compliant Mechanism	2023 - Sep	6,827	732	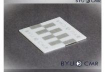	MaLO Hinge	2023 - Sep	811	35
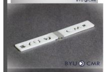	MaLDO Hinge	2023 - Sep	506	52	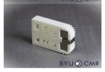	Magnetic Gear Links	2023 - Sep	815	44
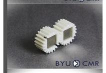	Magnetic Gears	2023 - Sep	1,108	102	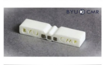	Bistable-to-Monostable Magnetic Hinge	2023 - Sep	747	30
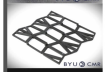	Volume-Efficient Miura-ori (VEMO) with windows	2023 - Aug	1,121	116	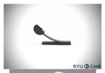	Euler Ctapult	2023 - Aug	1,259	172
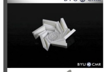	Folding Origami Flasher Hexagon with Living Hinges	2023 - Jul	5,128	520	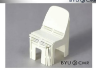	Single-Sheet Model Chair	2022 - Aug	1,722	184
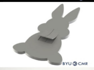	Self-Opening Easter Bunny	2022 - Apr	1,274	129	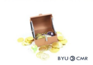	St. Patrick’s Day Treasure Chest	2022 - Mar	507	223
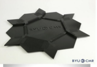	Folding Hexagon Origami Mechanism	2021 - Sep	1,142	189	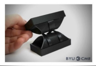	Developable Compliant Rolling-Contact Element (D-CORE)	2021 - Apr	2,443	1,029
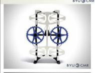	Linear-Motion Compliant Mechanism	2021 - Feb	436	324	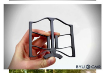	Large-Displacement Linear Motion Mechanisms	2021 - Jan	879	472
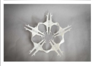	Kaleido-Flake	2020 - Dec	436	324	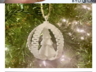	Christmas Ornament - Compliant LET Joints	2020 - Dec	797	1,023
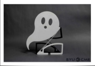	Ghost Cross-Axis Flexural Pivot	2020 - Oct	1,241	770	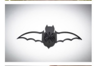	Bat-Flex	2020 - Oct	1,486	676
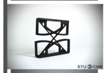	Cross-Axis Flexure Pivot	2020 - Oct	1,211	895	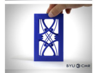	Constant-Force Mechanism	2020 - Oct	8,003	4,440
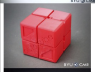	OctoCube	2019 - Oct	9,668	2,492	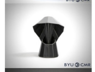	Elliptic Infinity	2019 - Oct	7,611	2,707
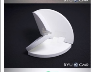	Four-Bar Origami Vertex	2019 - Oct	3,992	2,142	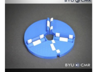	SOURCE Origami Vertex	2019 - Oct	3,164	1,297
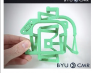	2 DOF Fully Compliant Space Pointing Mechanism	2019 - May	48,179	9,857	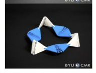	Kaliedocycle	2019 - Apr	7,806	2,022
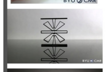	Flex-16: Large-Displacement Monolithic Compliant Rotational Hinge	2019 - Mar	9,361	2,698	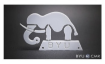	“Wyrd” Elephant Compliant Mechanism	2019 - Mar	57,455	17,795
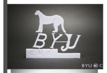	“Wyrd” Cougar Compliant Mechanism	2019 - Mar	429	19	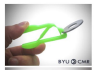	Fully Compliant Pliers	2019 - Mar	90,150	24,613
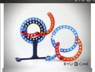	CurvedLinkns: Large Size Circular Links	2019 - Feb	4,645	1,297	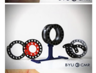	CurvedLinks: Medium Size Circular Links	2019 - Feb	3,511	1,225
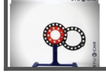	CurvedLinks: Small Size Circular Links	2019 - Feb	2,981	757	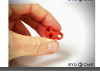	CurvedLinks: Adapter Piece	2019 - Feb	6,674	720

"Thing"	Date Published	Views	Downloads	"Thing"	Date Published	Views	Downloads
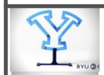 Morphing "Y": A One-DOF Six-bar Developable Mechanims	2019 - Feb	4,608	953	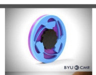 Compliant Overrunning Clutch	2018 - Nov	29,336	5,978
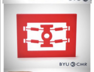 Bistable Compliant Mechanism	2018 - Nov	67,536	19,868	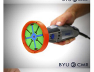 Compliant Centrifugal Clutch	2018 - Nov	34,825	5,107
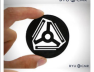 Orth-Planar Spring	2018 - Aug	14,085	3,728	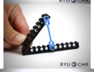 FlexLinks: Fixed-Fixed Beam	2018 - Jul	437	168
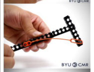 FlexLinks: Fixed-Slotted Beam	2018 - Jul	3,060	692	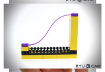 FlexLinks: Fixed-Fixed Beam - Horizontal Straight	2018 - Jul	695	103
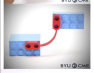 FlexLinks: Fixed-Fixed Beam - Quarter Circle	2018 - Jul	378	119	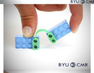 FlexLinks: Fixed-Fixed Beam - Half Circle	2018 - Jul	4,039	1,142
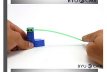 FlexLinks: Cantilever Beam	2018 - Jul	843	49	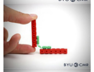 FlexLinks: Cantilever Beam Adjust	2018 - Jul	308	64
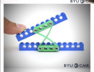 FlexLinks: Cross-Axis Flexural Pivot	2018 - Jul	4,165	688	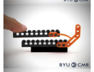 FlexLinks: "Satellite"	2018 - Jul	2,798	269
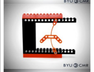 FlexLinks: "Airplane"	2018 - Jul	2,375	255	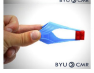 Oriceps: Origami Inspired Forceps	2018 - Jul	41,053	11,534
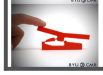 Bistable Compliant Switch	2018 - Jul	49,922	16,774				
					**Total:**	**912,645**	**245,861**

)

Supplemental Table 2: Total views statistics for the eight “Instructables” provided by Instructables analytics.

“Instructable		Date Published	Views	“Intructable”		Date Published	Views
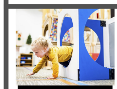	Hidey Cube (LET Joint)	2023 - Aug	941	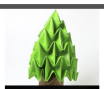	Origami Christmas Tree	2019 - Dec	2,102
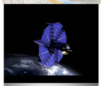	Origami Flasher	2019 - Oct	29,590	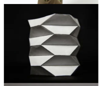	Origami Ballistic Barrier Paper Model	2019 - Oct	12,241
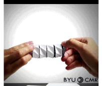	Color Changing Bidirectional Tube	2019 - Oct	21,881	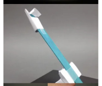	Catapult Origami	2019 - Apr	1,822
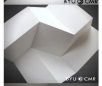	Origami Square Twist	2018 - Oct	16,835	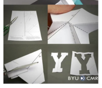	One Cut “Y”	2018 - Aug	4,032
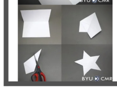	One Cut Origami Star	2018 - Jul	19,891				
						**Total:**	**109,335**
