# Supplementary material for: Expanding research impact through engaging the maker community and collaborating with digital content creators
Source: PLoS One. 2024 May 8;19(5):e0302449. doi: 10.1371/journal.pone.0302449 (PMC11078436; doi:10.1371/journal.pone.0302449)
Supplement: Attachment — Submitted filename: Response to Reviewers.pdf [file pone.0302449.s003.pdf]

Resolution to Review of “Expanding research impact through engaging the maker community and collaborating with digital content creators”

We thank the reviewers and editor for their thoughtful comments. We have carefully reviewed each comment and the paper has been modified accordingly. The text below lists each reviewer comment followed by our response and a summary of the changes made in response to the comment.

Journal requirements

Comment: We note that you have provided funding information that is not currently declared in your Funding Statement. However, funding information should not appear in the Acknowledgments section or other areas of your manuscript. We will only publish funding information present in the Funding Statement section of the online submission form. Please remove any funding-related text from the manuscript and let us know how you would like to update your Funding Statement… Please include your amended statements within your cover letter; we will change the online submission form on your behalf.

> *Author Action*: Funding information has been removed from the acknowledgments section. The Funding Statement contains a full declaration of funding information.

Comment: Please ensure that your manuscript meets PLOS ONE's style requirements, including those for file naming. The PLOS ONE style templates can be found at https://journals.plos.org/plosone/s/file?id=wjVg/PLOSOne_formatting_sample_main_body.pdf and https://journals.plos.org/plosone/s/file?id=ba62/PLOSOne_formatting_sample_title_authors_affiliations.pdf.

> *Author Action*: The manuscript uses the PLOS ONE LaTeX template which helps the body meet style requirements. The title, authors, and affiliation also matches style guides when compared to recently published articles from PLOS ONE.

Comment: Please include captions for your Supporting Information files at the end of your manuscript, and update any in-text citations to match accordingly. Please see our Supporting Information guidelines for more information: http://journals.plos.org/plosone/s/supporting-information.

> *Author Action:* Captions have been added for the Supporting Information files at the end of the manuscript. In-text citations have also been updated to reference this according to the provided guidelines.

Comment: Please review your reference list to ensure that it is complete and correct. If you have cited papers that have been retracted, please include the rationale for doing so in the manuscript text, or remove these references and replace them with relevant current references. Any changes to the reference list should be mentioned in the rebuttal letter that accompanies your revised manuscript. If you need to cite a retracted article, indicate the article’s retracted status in the References list and also include a citation and full reference for the retraction notice.

> *Author Action:* The reference list has been double-checked for completion and correctness. No retracted articles are cited. References 1, 26-28, 34, 46, 51, 54, and 57 had information added to the reference and/or the website links in the reference were modified appropriately. Reference 15 was removed from the citation list because it was deemed unnecessary.

Reviewer #1

Comment: This article proposes a method to increase the impact of academic research by making the material available for public use, thereby engaging the maker community and collaborating with internet content creators to expand reach. This is an approads intersting. The research process took place over four years with short-term and long-term data collection, showing that data collection was taken seriously. The research results are analyzed in detail. The article is suitable for publication.

Author Response: Thank you for your review.

Reviewer #2 (should we thank him in the acknowledgments section?)

Comment: The paper reports on a hugely successful initiative to engage the maker community with research by professional and academic engineers. The authors set out a framework for others to follow in order to emulate their success. I thought the paper was well written, well founded and the display items were neatly presented. I fully agree with the sentiment of the paper that there's a mutual benefit to be had by pushing at what's effectively an open door with an already interested public. I do have some comments and suggestions that I set out below for consideration by the authors.

Author Response: Thank you.

Comment: Figure 1. Here you set out your linear framework for dissemination, but I wonder if this speaks to an older form of science communication (the deficit model) that doesn't accurately reflect what you found. I could see the figure working better as a cycle because in engaging with the community you're seeding new academic researchers who will conduct new research and start the cycle over. Thus, it's far more participatory. See 'Trench, B. (2008). Towards an analytical framework of science communication models. Communicating science in social contexts: New models, new practices, 119-135.'

Author Response: Our approach to the framework was indeed cyclical, so a cyclical model would be a better representation.

Author Action: We changed fig. 1 into a figure depicting a cyclical process rather than a linear process.

Comment: With a view of how this could be extended to other research communities, I found parts of the paper quite vague:
1. Line 187 you write: "Considering the turnover in a typical university research lab, tutorials and templates were created to outline a repeatable and sustainable process to help the next generation of students continue supporting the ongoing outreach strategy" but what are these tutorials and templates about?
2. Line 199 you write how "we identified and collaborated with content creators who (1) were adequately informed in their area of research, (2) were engaging and well-received by the desired community, (3) had a substantial following that reflected the target audience demographics, and (4) had a reputation that aligned with that of the research group." None of these are defined, what's a substantial following for example. Is there a threshold for this? How do you assess someone's reputation? All valuable information for your reader. Clearly, the content creators are incredibly popular and fit these criteria but, as it stands, this sounds like a post hoc rationalization rather than something others could use.

Author Response: 1. Tutorials and templates were created regarding the process of how to conduct research, upload prototypes to makerspaces, post on social media, and engage with the maker community.

2. These items are hard to assess because social media and platforms that we use for outreach are constantly changing as well as the expectations of an influencer.

Author Action: 1. We edited the language in the "Tutorials to facilitate sustainability" section to further clarify the actions taken.

2. To solve this problem we kept the same basic points but specified them to point out specifically the description of the content creators we collaborated with, then mentioned how one or multiple of those attributes are desirable for outreach work in any field of study.

Comment: Methods – please add some times of the project interventions e.g. the dates the YouTube videos of the content creators went live.

Author Response: This addition can be helpful to the reader.

Author Action: We added specific dates each YouTube video was posted as well as mentioning that we periodically posted our own content on maker platforms before and after collaborating with influencers.

Comment: In light of open science principles, are the data from the surveys and code for your figures available somewhere?

Author Response: They are.

Author Action: A zip file with data from surveys was added to the submission.

Comment: Line 140 - For the models you created you list some considerations that would make them more likely to be adopted by the maker community. Did you measure which of the considerations each of the objects met? Perhaps those that met specific criteria were more likely to be used - something you could test statistically.

Author Response: This is a good idea and would be valuable to future work.

Author Action: We added a sentence after the considerations: For future studies, it would be beneficial to measure which of these considerations made a model more attractive to the maker community.

Comment: At some points I found your use of the passive voice a bit confusing because it masked who was responsible for the action. E.g. "At the time of writing, fifty-six 3D printable designs have been uploaded to maker repositories (Thingiverse and Printables) and nine projects have been uploaded to Instructables. Six examples of 3D printable models uploaded to Thingiverse and Printables are shown in Fig 3."

Author Response: The use of passive voice does occasionally obscure who performed the action.

Author Action: We updated the quote in question, as well as several quotes which had similar issues, to clarify who performed the actions. The quote now reads:

At the time of writing, we have uploaded fifty-six 3D printable designs to maker repositories (Thingiverse and Printables) and nine projects to Instructables. Six examples of these 3D printable models are shown in Fig 3. To support the model, we also uploaded lesson plans, descriptions, and short YouTube videos which complement the uploaded content.

An additional quote that was edited now reads: In 2019, we identified and contacted several content creators with potential for collaboration.

Comment: You focus your data analysis on descriptive statistics rather than anything inferential, so you're ultimately left with correlations between your initiatives and the various measures of popularity. Though it's clear your work was driving these patterns I think you could consider discussing how to quantify your impact. Some recent papers describe how to do so e.g. the causal impact framework Brodersen KH, Gallusser F, Koehler J, Remy N, Scott SL. Inferring causal impact using Bayesian structural time-series models. Annals of Applied Statistics, 2015, Vol. 9, No. 1, 247-274. http://research.google.com/pubs/pub41854.html Just to be clear, I'm not hinging my review on this point but it should be acknowledged especially given the confound that COVID may have had on some these findings with an eager maker community stuck at home and looking for content.

Author Response: The correlation rather than causation is an important point. It is also important to bring up COVID as a confounding factor.

Author Action: We added a sentence to the "Repeated Study" section: It is also important to note here that COVID-19, which began to initiate international lockdowns in early 2020, significantly increased the activity of people looking for content, including makers looking for models to create. This trend towards making has continued through the ensuing four years. This may be a factor influencing the greater numbers seen following the second video.

Comment: Figure 11. is the order the wrong way around here? Panel (c) looks like the Lego-Compatible device not panel (d). And when I check out this site (https://compliantmechanisms.byu.edu/maker-resources) that you refer to earlier panel (d) looks like a 'One-Piece Compliant Disk Launcher'

Author Response: Thank you for catching this and for your extra work to verify the issue.

Author Action: The order of the mechanisms listed was corrected and some spelling modified.

Comment: In the discussion and even the introduction I think you could offer your perspective on how the framework could be expanded to other areas in STEM. Are there some fields where it's easy to apply (biology/ palaeontology, which is my own area, seems like an obvious one – imagine having a 3D model of a new dinosaur fossil made available)? Or is there something unique about the maker community? Further, you don't mention citizen science in your work, but it's intimately related to these ideas.

Author Response: The applicability of the research to other areas is important to mention.

Author Action: Weadded some text discussing how this framework is applicable to other areas in STEM, including some discussion on citizen scientists.

Comment: More could be made of the limitations / future directions of the work. Is this type of framework applicable to other countries? Is there a gender component? Are we beholden to hugely popular content creators to catalyze dissemination? How do we encourage more people to take on these sorts of roles when academia is so invested in prioritizing research funding and publications over engagement? I'm conscious that you can't consider all of these points, but they all came to mind as I was reading the piece.

Author Response: Discussion of limitations and future directions is also important to consider.

Author Action: Several of these points were added to the Introduction and Discussion sections, including that this framework is likely highly applicable to other fields and other countries and that content creators don't always need to be the ones to disseminate information. Additionally, a figure showing gender disparity of survey respondents and a discussion on this

figure were added to the Outcomes. Finally, an emphasis on the international reception of our outreach efforts was added.

Comment: Some of the references are missing links to the relevant source e.g. 'Green T. Publication is not enough, to generate impact you need to campaign. Impact of Social Sciences. 2019.'

Author Response: True.

Author Action: We checked each reference individually, added information where possible, and deleted reference 15 after evaluating it and realizing it was not necessary.

Comment: On a final note, I watched both videos and now know what compliant mechanisms are, so you've got through to another person!

Author Response: Thank you!

Reviewer #3

Comment: The manuscript is technically sound, and the data support the conclusions.
The statistical analysis has been performed appropriately and rigorously.
The authors have made all data underlying the findings in their manuscript fully available.
The manuscript is presented in an intelligible fashion and written in standard English.

Author Response: Thank you for your review.
